# Case of an unusual clinical and radiological presentation of pulmonary metastasis from a costal chondrosarcoma after wide surgical resection: A transbronchial biopsy is recommended

**DOI:** 10.1186/1477-7819-9-50

**Published:** 2011-05-16

**Authors:** Makoto Emori, Ken-ichiro Hamada, Takenori Kozuka, Katsuyuki Nakanishi, Yasuhiko Tomita, Norifumi Naka, Nobuhito Araki

**Affiliations:** 1 Department of Orthopedic Surgery, Osaka Medical Center for Cancer and Cardiovascular Diseases, Osaka 537-8511, Japan; 2 Department of Radiology, Osaka Medical Center for Cancer and Cardiovascular Diseases, Osaka 537-8511, Japan; 3 Department of Pathology and Cytology, Osaka Medical Center for Cancer and Cardiovascular Diseases, Osaka 537-8511, Japan; 4 Department of Orthopedic Surgery, Sapporo Medical University School of Medicine, Hokkaido 060-8556, Japan

## Abstract

Chondrosarcomas are the most frequently occurring primary malignant chest wall tumors. Furthermore, the lungs serve as the most frequent sites for metastases. Pulmonary metastases from sarcomas usually appear as round nodules of varying sizes on roentgenograms. Here, we report an unusual clinical and radiographic presentation of pulmonary metastasis from a costal chondrosarcoma. Bilateral pulmonary metastases developed soon after wide surgical resection. Thoracic computed tomography revealed unusual radiological findings: consolidation accompanied with ground-glass opacity. To confirm the metastasis, we recommend a transbronchial biopsy in cases where unusual pulmonary findings are detected.

## Background

Chondrosarcomas are the second most frequent primary malignant bone tumors, after osteosarcomas [1,2]. They are also the most common primary malignant chest wall tumors: 5-15% of chondrosarcomas are located in the thoracic wall [3]. Since radiotherapy and chemotherapy are generally ineffective against chondrosarcomas, surgery is the only curative treatment, and the quality of the surgery is an essential prognostic factor [2]. Enneking et al. classified surgical margins into wide, marginal, and intralesional [4]. A wide resection is accomplished by a procedure in which the lesion, its pseudocapsule and/or reactive zone, and a surrounding cuff of normal tissue are taken as a single block. Therefore, resection for chest wall chondrosarcoma should be wide, taking intact pleura internally, intact muscle fascia externally, and transverse rib resection > 2 cm from the tumor on both directions [4,5]. Clinically, the involved rib en bloc should be resected along with the 2 intercostal spaces above and below the tumor.

On roentgenograms, pulmonary metastases usually appear as multiple peripheral, round nodules of varying sizes. Here, we describe an atypical presentation of pulmonary metastasis occurring soon after wide surgical resection of a costal chondrosarcoma. In this case, a thoracic computed tomography (CT) scan showed consolidation, predominantly in both the lower lobes, surrounded by ground-glass opacities and air bronchograms, mimicking serious pneumonia.

## Case presentation

**Figure 1:**
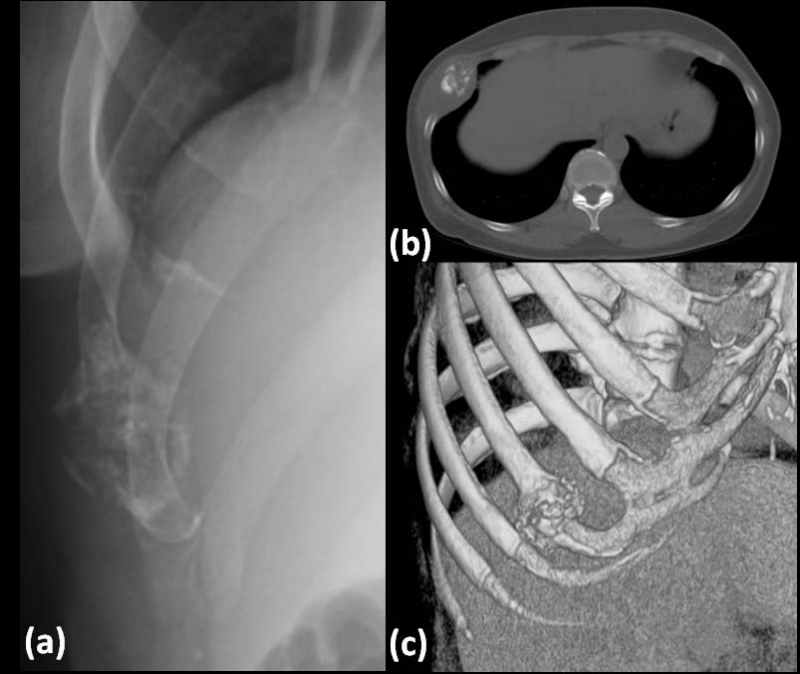
***Preoperative radiological examinations***. *(a) X-ray showing a mass with coarse calcification located in the right eighth rib, expanding beyond the irregular cortex. (b) CT scan showing a low -density mass with coarse calcification along the right eighth rib; the mass arose at the bone-cartilage border. (c) 3D-CT scan showing destruction of bone and cartilage destruction, with expansive growth of the tumor at the right eighth rib*.

**Figure 2:**
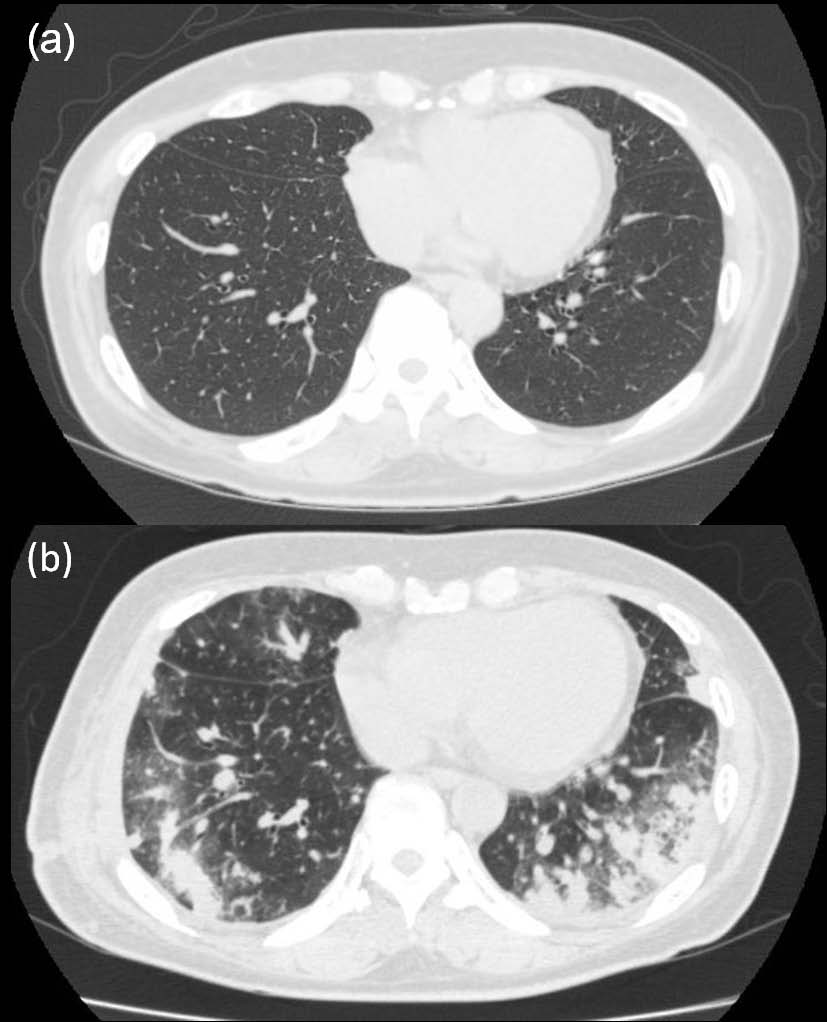
***Chest CT scan***. *(a) Preoperative CT scan showing no pulmonary metastasis. (b) Postoperative CT scan showing pulmonary non-segmental consolidation, predominantly in the peripheral lung field, with surrounding ground-glass opacities; no bronchovascular bundle thickness or interlobular septal thickness was observed*. The tumor was resected together with the right seventh, eighth, and ninth ribs.

**Figure 3:**
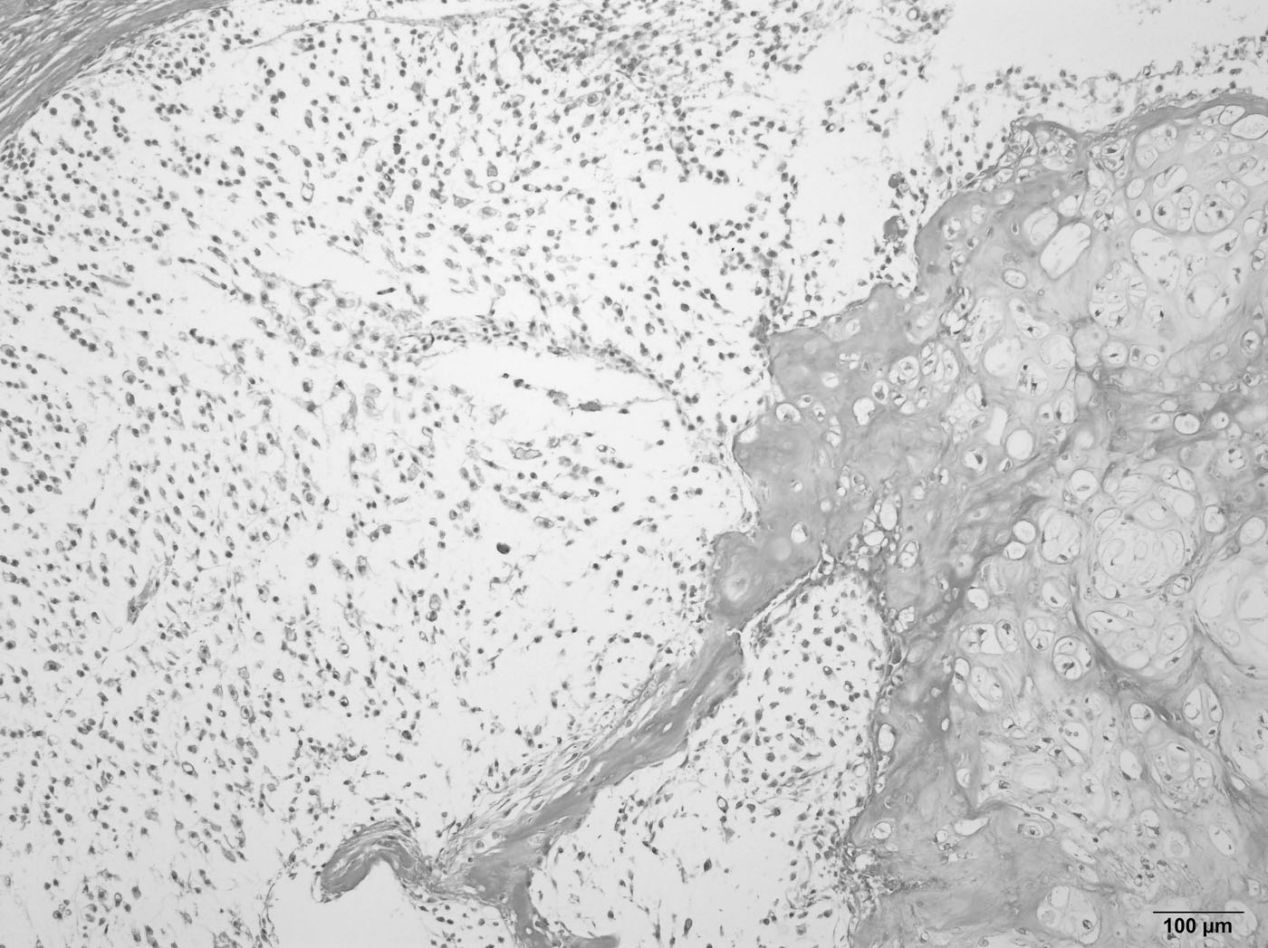
***Resected tumor specimen***. *Hematoxylin and eosin staining of the resected tumor showed a mild increase in cellularity and nuclear atypia. Doubly nucleated cells were seen in the field*.

A 62-year-old woman was admitted to our hospital because of a mass that grew gradually in the right lateral chest wall for 1 year. Physical examination revealed a tumor (5 × 3.5 cm) in the right eighth rib. The mass was hard with an unclear border, no mobility, redness, or local heat, but it was tender. An X-ray revealed a mass with coarse calcification located on the right eighth rib, expanding beyond the irregular cortex. Thoracic CT revealed a 70 × 60 × 30 mm low-density mass (CT value, +18 HU) along the right eighth rib; it arose at the bone-cartilage border and destroyed these tissues (Figure 1a-c). No pulmonary metastasis was observed (Figure 2a). Other metastatic workup, including PET scan, was negative. The physical examination and imaging findings strongly indicated primary chondrosarcoma. Therefore, wide surgical resection was performed without performing a biopsy; the tumor was resected together with the right seventh, eighth, and ninth ribs. Transverse rib resection was performed >4 cm from the tumor in both directions. The chest wall was reconstructed using a Dexon mesh^® ^(US Surgical, Connecticut, USA). Histological examination revealed a grade II chondrosarcoma with increased cellularity and myxoid stroma (Figure 3). All resected surgical margins were wide. The postoperative course was uneventful, and the patient was discharged 2 weeks after the operation.

**Figure 4:**
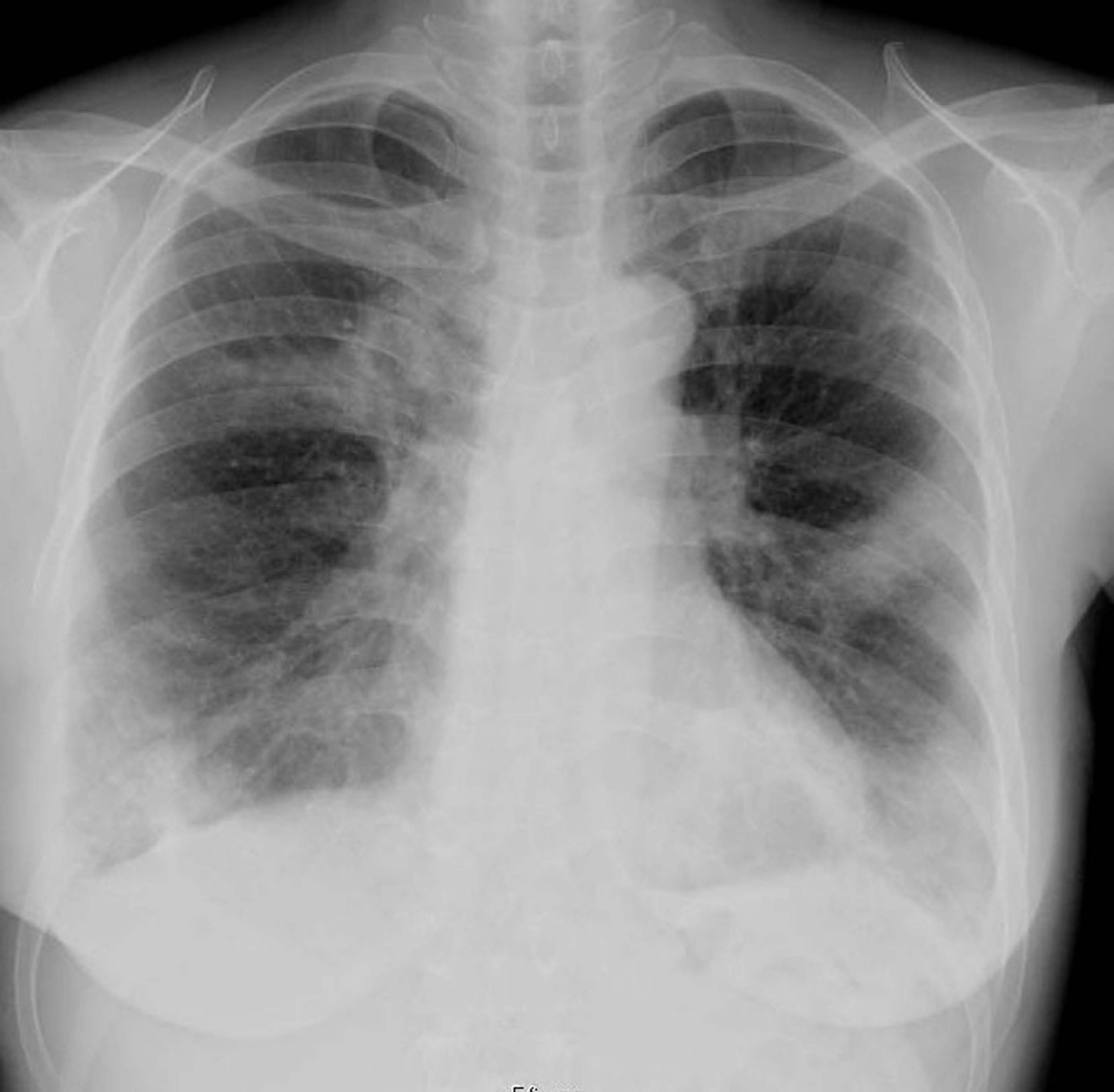
***Chest X-ray***. *Chest roentgenogram showed air-space consolidation with an air bronchogram, predominantly in the right upper and left lower lung fields*.

**Figure 5:**
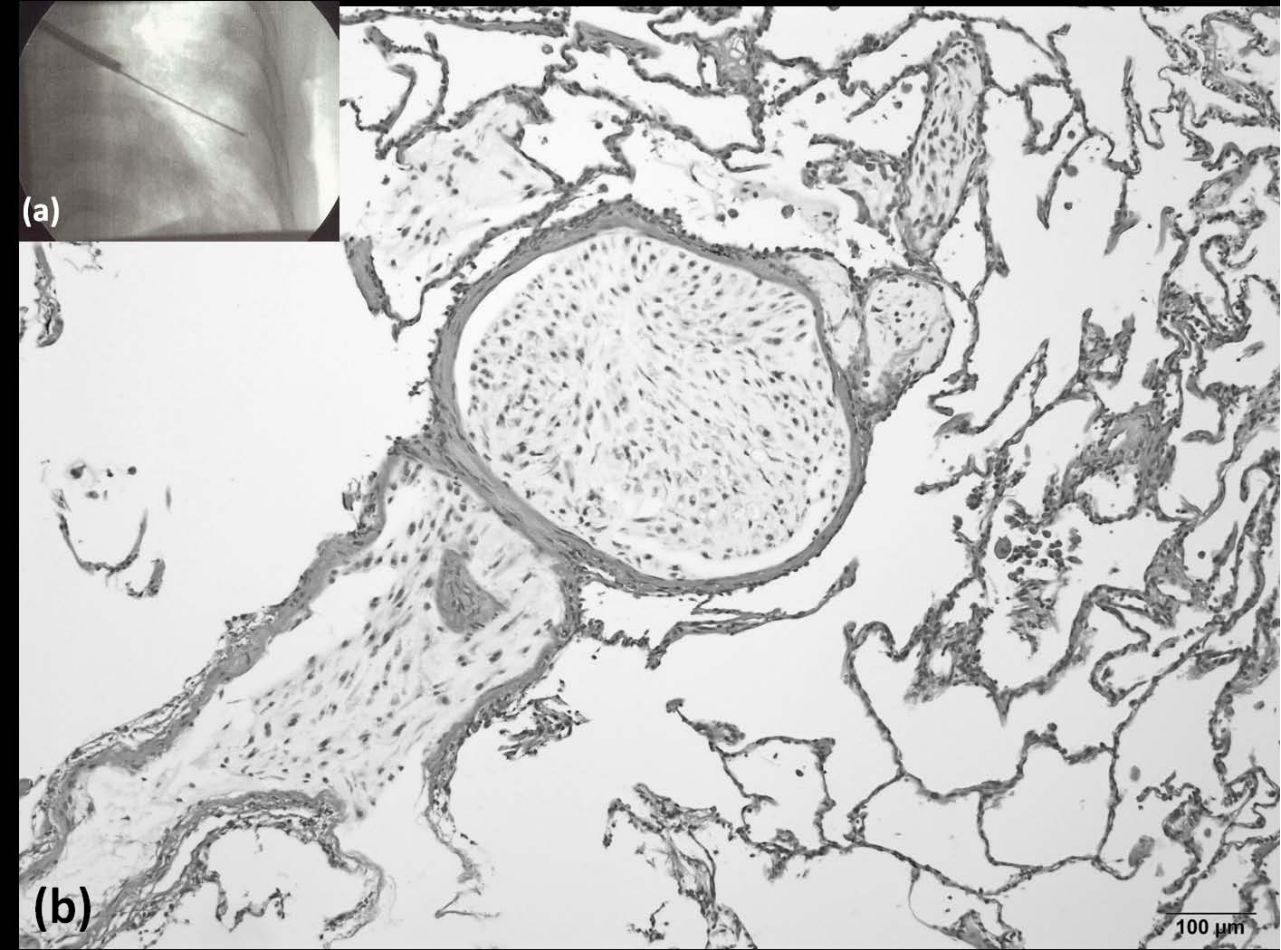
***Bronchoscopy***. (a) Transbronchial biopsy was performed through the left S8 bronchus. *(b) Hematoxylin and eosin staining of the biopsy sample showed a bone tumor in the lumen structure, with the same histological features as the primary bone tumor*.

However, 7 weeks after the definitive surgery, she presented with a slight fever, dyspnea, persistent dry cough, and purulent nasal discharge of 1-week duration. The white blood cell count (WBC)and C-reactive protein (CRP) level were 8.2 × 10^9 ^cells/L (neutrophils, 75%; lymphocytes, 16%; monocytes, 4.7%) and 3.7 mg/dL (normal: <0.30 mg/dL) respectively. Findings of other biochemical and serologic tests were normal. The chest roentgenogram showed air-space consolidation accompanied with an air bronchogram in the right upper and left lower lung fields (Figure 4) - a finding highly suggestive of bacterial pneumonia. Antibiotics (tazobactam/piperacillin [TAZ/PIPC]) administered for 7 days showed no results. Thoracic CT revealed pulmonary non-segmental consolidation, predominantly in the peripheral lung field, surrounded by ground-glass opacities; bronchovascular bundle thickness and interlobular septal thickness were absent (Figure 2b). Bronchoscopy and consequent transbronchial biopsy revealed blood vessel proliferation in the bronchial wall. Therefore, we considered this as a case of interstitial pneumonia such as cryptogenic organizing pneumonia, and initiated glucocorticoid therapy without waiting for the biopsy results. However, 3 days after the onset of the treatment, transbronchial biopsy sample through the left S8 bronchus confirmed the same histological features as the primary tumor in the peritumoral lumen structure, which was negative for CD34 and D2-40 (Figure 5a, b). The bronchoalveolar lavage fluid culture was negative. The patient died 12 weeks after the definitive surgery.

## Discussion

Chondrosarcomas are classified on the basis of their aggressiveness into 3 grades according to their cellular density, degree of anisokaryosis, and nuclear hyperchromatism [6]. The histologic grades of chondrosarcoma correlate well with prognosis, especially for metastases [6]. The most frequent site of metastasis is the lungs; other sites include the bones, brain, regional lymph nodes, and liver [5]. The metastasis rates for grades I, II, and III tumors were 0, 13, and 23%, respectively [5]. The incidence of pulmonary metastases varies with the primary tumor and stage of disease. Bone tumors such as osteosarcomas and Ewing's sarcoma show a high incidence of pulmonary metastases. Pulmonary metastasis develops from 20% of the chondrosarcomas of the chest wall [5]. The most common route for pulmonary metastasis of sarcomas is hematogenous dissemination; therefore, most pulmonary metastases appear as multiple peripheral, round nodules of varying sizes on roentgenograms. However, certain sarcomas such as osteosarcomas present with unusual features of pulmonary metastasis, i.e., lymphangitic carcinomatosis, endobronchial metastasis, or pneumothorax [7,8].

The pulmonary metastasis in this case was atypical in the following ways: (1) The radiological features mimicked those of pneumonia. Thoracic CT revealed pulmonary non-segmental consolidation, predominantly in the peripheral lung field, surrounded by ground-glass opacities. This indicated interstitial pneumonia such as cryptogenic organizing pneumonia. (2) Although the operation involved only the right side, bilateral pulmonary metastases developed after the resection. Time taken for metastasis to develop has been reported to be an average of 20 months [2]. In this case, bilateral pulmonary lesions rapidly developed into metastases. Thus, histologic examination was needed in order to confirm the diagnosis.

Transbronchial biopsy, endobronchial biopsy, or surgical lung biopsy can be performed to obtain tissue specimens. Surgical lung biopsy includes video-assisted thoracic surgery (VATS) and open lung biopsy. The procedure chosen is based on clinical judgment, which entails weighing the yield versus the risk to the patient. In particular, transbronchial biopsy is usually the procedure of choice for the initial examination due to its high yield and relatively low risk [9], and therefore, we chose this approach. The transbronchial biopsy revealed pulmonary metastasis from costal chondrosarcoma although the mechanism underlying the pulmonary metastasis remains unknown. The possibility of lymphangitic carcinomatosis was eliminated because of the absence interlobular septal thickness.

Soon after the curative surgery is performed, to confirm the pulmonary metastasis, we recommend that transbronchial biopsy should be performed in cases where unusual clinical and radiological pulmonary findings are detected.

## Informed consent

Written informed consent was obtained from the patient for publication of this case report and accompanying images. A copy of the written consent is available for review by the Editor-in-chief of this journal.

## Authors' contributions

ME: assisted in the writing of the manuscript and in the orthopedic workup of the patient; KH: assisted in the drafting of the manuscript and in the orthopedic workup of the patient; TK: assisted in the writing of the manuscript and performed the radiological evaluation; KN: performed the radiological evaluation; YT: performed the pathological evaluation; NN: assisted in the orthopedic workup of the patient; NA: evaluated critically the manuscript and gave final approval for the manuscript to be published. All authors read and approved the final manuscript.

## Competing interests

The authors declare that they have no competing interests.
